# Predictors of severity and mortality in COVID-19 patients

**DOI:** 10.1186/s43168-022-00122-0

**Published:** 2022-03-26

**Authors:** Hebatallah Hany Assal, Hoda M. Abdel-hamid, Sally Magdy, Maged Salah, Asmaa Ali, Rasha Helmy Elkaffas, Irene Mohamed Sabry

**Affiliations:** 1 Department of Chest Medicine, Faculty of Medicine, Cairo University, Al Kasr Al Aini, Old Cairo, Cairo, 11956 Egypt; 2 Department of Anaethesia, Faculty of Medicine, Cairo University, Cairo, Egypt; 3 Department of Chest, Abbassia Chest Hospital, Ministry of Health and Population (MOHP), Cairo, Egypt; 4 Department of Clinical and Chemical Pathology, Faculty of Medicine, Cairo University, Cairo, Egypt

**Keywords:** COVID-19, Severity, Mortality, SARS-CoV-2

## Abstract

**Background:**

Due to limited capacity, health care systems worldwide have been put in challenging situations since the emergence of COVID-19. To prioritize patients who need hospital admission, a better understanding of the clinical predictors of disease severity is required. In the current study, we investigated the predictors of mortality and severity of illness in COVID-19 from a single center in Cairo, Egypt.

**Methods:**

This retrospective cohort study included 175 patients hospitalized with COVID-19 pneumonia and had positive real-time polymerase chain reaction (RT-PCR) results for SARS-CoV-2 from 1 May 2020 to 1 December 2020. Severe COVID-19 was defined as requiring high-flow oxygen (flow rate of more than 8 L/min or use of high flow oxygen cannula), noninvasive ventilation, or invasive mechanical ventilation at any time point during the hospitalization. We used univariate and multivariate regression analyses to examine the differences in patient demographics and clinical and laboratory data collected during the first 24 h of hospitalization related to severe disease or death in all 175 patients.

**Results:**

Sixty-seven (38.3%) of the study subjects had a severe or critical disease. Elevated d-dimer, leukocytosis, and elevated CRP were found to be independent predictors of severe disease. In-hospital mortality occurred in 34 (19.4%) of the cases. Elevated TLC, urea, the use of invasive mechanical ventilation, and the presence of respiratory bacterial co-infection were found to be independently associated with mortality.

**Conclusion:**

Clinical and laboratory data of COVID-19 patients at their hospital admission may aid clinicians in the early identification and triage of high-risk patients.

## Introduction

The SARS-CoV-2 pandemic began in Wuhan, China, in December 2019, and continues to pose a challenge to the healthcare system worldwide [1]. According to the WHO’s most recent update, there have been over 255 million cases worldwide, with over 5 million deaths reported.

Since the emergence of the SARS-CoV-2 in late 2019 and the WHO’s official declaration of a worldwide pandemic in March 2020, there have been tremendous efforts to identify prognosticators that clinicians utilize to assess the risk at the early stage of the disease, thus aiding in tailoring management strategies as well as facilitating decision-making and improving outcomes for COVID-19 patients via increasing the cure rate and decreasing the case fatality rate.

Despite ongoing research, the clinical characteristics and outcomes of COVID-19 patients as a population have yet to be thoroughly studied in Egypt.

We conducted a retrospective cohort study of COVID-19 patients hospitalized in an Egyptian tertiary hospital to determine the risk factors associated with a severe course and worse outcomes, including mortality, to assist healthcare systems in triaging patients who present to the hospital.

## Methods

### Study subjects and settings

We performed a retrospective cohort study of 175 patients hospitalized with COVID-19 from 1 May 2020 to 1 December 2020 in the quarantine section of Misr International Hospital, Cairo, Egypt.

Patients who were hospitalized with radiological evidence of COVID-19 pneumonia and had positive real-time polymerase chain reaction (RT-PCR) results for SARS-CoV-2 were included in the study. Patients with missing data and negative (RT-PCR) results for SARS-CoV-2 were excluded from the study.

### Data collection

Demographic and laboratory data were extracted from the medical records. The following clinical data were collected: baseline comorbidities, presenting symptoms, vital signs, microbiology results, imaging results, medical treatments, supplemental oxygen (O_2_), noninvasive and invasive forms of ventilation, respiratory co-infections, complications, and hospitalization outcome (death or discharge).

### Ethics approval

The institutional review board of the Ministry of Health, Cairo, Egypt (No: 3- 2021/19) approved the study. The data was collected from the hospital records, and informed consent was not required as the data was anonymized and no personal identifiers were collected.

### Patient categorization

We defined severe COVID-19 as requiring high-flow O_2_ (flow rate of more than 8 L/min or use of high-flow oxygen cannula), noninvasive ventilation, or invasive mechanical ventilation at any time point during the hospitalization. Among all 175 patients, we investigated the differences in the demographic, clinical, and laboratory data collected during the first 24 h of hospitalization regarding severe disease or death at any time during hospitalization.

### Statistical methods

The data was collected and tabulated for statistical analysis using Minitab 17.1.0.0 for Windows (Minitab Inc., 2013, PA, USA). Continuous data were presented as mean and standard deviation (SD), whereas categorical data were presented as number and percentage (%). The normality of data was examined using the Shapiro-Wilk test. The association between severity and mortality was performed using the chi-square test, independent *t*-test, or Mann-Whitney test. Moreover, the prognostic utility of TLC, d-dimmer, urea, and CRP was done using the receiver operating characteristic curve (ROC curve); the area under the curve AUC above 0.6 is considered acceptable for test capability. Multiple logistic regression analysis models with the step forward selection model technique were used for finding the predictors for COVID-19 severity and mortality. All tests were two-sided; *P*-value was considered significant if < 0.05.

## Results

A total of 175 patients were included in the study, with an average age of 59 years, and the majority were males (77.7%). 54.9% of the patients were comorbid; DM and HTN were the most frequently encountered comorbidities, as reported in one-third of the cases. Among cases with COVID-19 pneumonia, 38.3% had a severe and critical disease. In-hospital mortality occurred in 19.4% of the cases. More than half of the cases (62.9%) needed supplemental oxygen. Standard oxygen supply (low-flow nasal cannula, standard oxygen mask) was the most frequently used (52%). High-flow nasal cannula (HFNC) and noninvasive and invasive mechanical ventilation were used in 18.3%, 8.6%, and 20.6% of patients, respectively (Table 1).

**Table 1:** Clinical and demographic characteristics and the outcome of patients with COVID-19

Demographic character	
Age^c^	58.87 ± 14.1
Sex	
Female^a^	39 (22.3%)
Male^a^	136 (77.7%)
Comorbidity^a^	96 (54.9%)
DM^a^	63 (36%)
HTN^a^	63 (36%)
IHD^a^	8 (4.6%)
Malignancy^a^	7 (4%)
Renal disease^a^	13 (7.43%)
Severe/critical^a^	67 (38.3%)
Oxygen use^a^	110 (62.9%)
Standard oxygen^a^	91 (52%)
HFNC^a^	32 (18.3%)
NIV^a^	15 (8.6%)
IMV^a^	36 (20.6%)
Admission labs	
TLC (× 10^3^/μl)^b^	10.6 (7.3–15.1)
Lymphocyte (/μl)^b^	1485 (1044–2190)
HB (g/dl)^c^	13.187 ± 1.932
PLT (× 10^3^/μl)^b^	302 (227–387)
AST (U/l)^b^	44 (31–70)
ALT (U/l)^b^	54 (34–90)
Urea (mg/dl)^b^	54 (41–101)
Creatine (mg/dl)^b^	1 (0.9–1.5)
d-dimer (μg/ml)^b^	1008 (523–2963)
Ferritin (ng/ml)^b^	1036 (546–2332)
CRP (mg/l)^b^	92.8 (41.8–196.2)
Respiratory co-infection^a^	19 (10.9%)
In-hospital mortality^a^	34 (19.4%)

*DM* Diabetes mellitus, *HTN* Hypertension, *IHD* Ischemic heart disease, *HFNC* High-flow nasal cannula, *NIV* Noninvasive ventilation, *IMV* Invasive mechanical ventilation, *TLC* Total leukocytic count, *Hb* Hemoglobin, *PLT* Platelets, *AST* Aspartate aminotransferase, *ALT* Alanine transaminase, *CRP* C-reactive protein

^a^Data are represented as number and percentage

^b^Data are represented as median and interquartile range

^c^Data are represented as mean and standard deviation

### Factors associated with the severity of COVID-19 pneumonia

Sixty-seven subjects (38.2%) were categorized as severe COVID pneumonia among the studied group. Comorbidities (diabetes, hypertension, and renal impairment) were significantly associated with the severe COVID pneumonia group versus the non-severe group (Table 2).

**Table 2:** Univariate Cox analysis of the risk factors for the severity of COVID-19

Variable	Moderate (*n* = 108)	Severe/critical (*n* = 67)	*P*
Demographic character			
Age^a^	58 ± 14	60.3 ± 14.3	0.3^**ƪ**^
Sex			
Female^a^	26 (24%)	13 (19.4%)	0.46^$^
Male^a^	82 (75.9%)	54 (80.6%)	
Comorbidity^a^	47 (43.5%)	49 (73.1%)	**< 0.001**^**$**^
DM^a^	32 (32.6%)	31 (46.3%)	**0.02**^**$**^
HTN^a^	31 (28.7%)	32 (47.8%)	**0.01**^**$**^
IHD^a^	3 (2.8%)	5 (7.5%)	0.15^$^
Malignancy^a^	3 (2.8%)	4 (5.9%)	0.31^$^
Renal^a^	3 (2.8%)	10 (14.9%)	**0.001**^**$**^
Admission labs			
TLC (× 10^3^/μl)^b^	9.55 (6.7–12.5)	15.00 (10–18.8)	**< 0.001**^**§**^
Lymphocyte (/μl)^b^	1611 (1259–2168)	1281 (846–2255)	**< 0.001**^**§**^
HB (g/dl)^c^	13.32 ± 1.88	12.97 ± 2	0.12^**ƪ**^
PLT (× 10^3^/μl)^b^	299 (230–376)	304 (217–414)	0.76^**§**^
AST (U/l)^b^	39 (25–52)	57 (38–85)	**< 0.001**^**§**^
ALT (U/l)^b^	46 (29–77)	65 (43–106)	**< 0.001**^**§**^
Urea (mg/dl)^b^	47 (36–72.5)	66 (49–167)	**< 0.001**^**§**^
Creatine (mg/dl)^b^	1 (0.8–1.2)	1.1 (0.9–1.9)	0.11^**§**^
d-dimer (μg/ml)^b^	680 (427–1398)	2216.64 (908–6830)	**< 0.001**^**§**^
Ferritin (ng/dl)^b^	794 (376–1415)	2198 (890–2645)	**< 0.001**^**§**^
CRP (mg/l)^b^	74.60 (31.7–126)	173.8 (80.4–263.3)	**< 0.001**^**§**^

*DM* Diabetes mellitus, *HTN* Hypertension, *IHD* Ischemic heart disease, *TLC* Total leukocytic count, *Hb* Hemoglobin, *PLT* Platelets, *AST* Aspartate aminotransferase, *ALT* Alanine transaminase, *CRP* C-reactive protein

^ƪ^Independent *t*-test

^§^Mann-Whitney test

^$^Chi-square test, *P* < 0.05 considered significant

^a^Data are represented as number and percentage

^b^Data are represented as median and interquartile range

^c^Data are represented as mean and standard deviation

Patients with severe COVID pneumonia had significantly higher levels of leukocytosis, lymphopenia, elevated liver enzymes, urea, d-dimer, ferritin, and CRP compared to the non-severe group (Table 2).

**Fig. 1:**
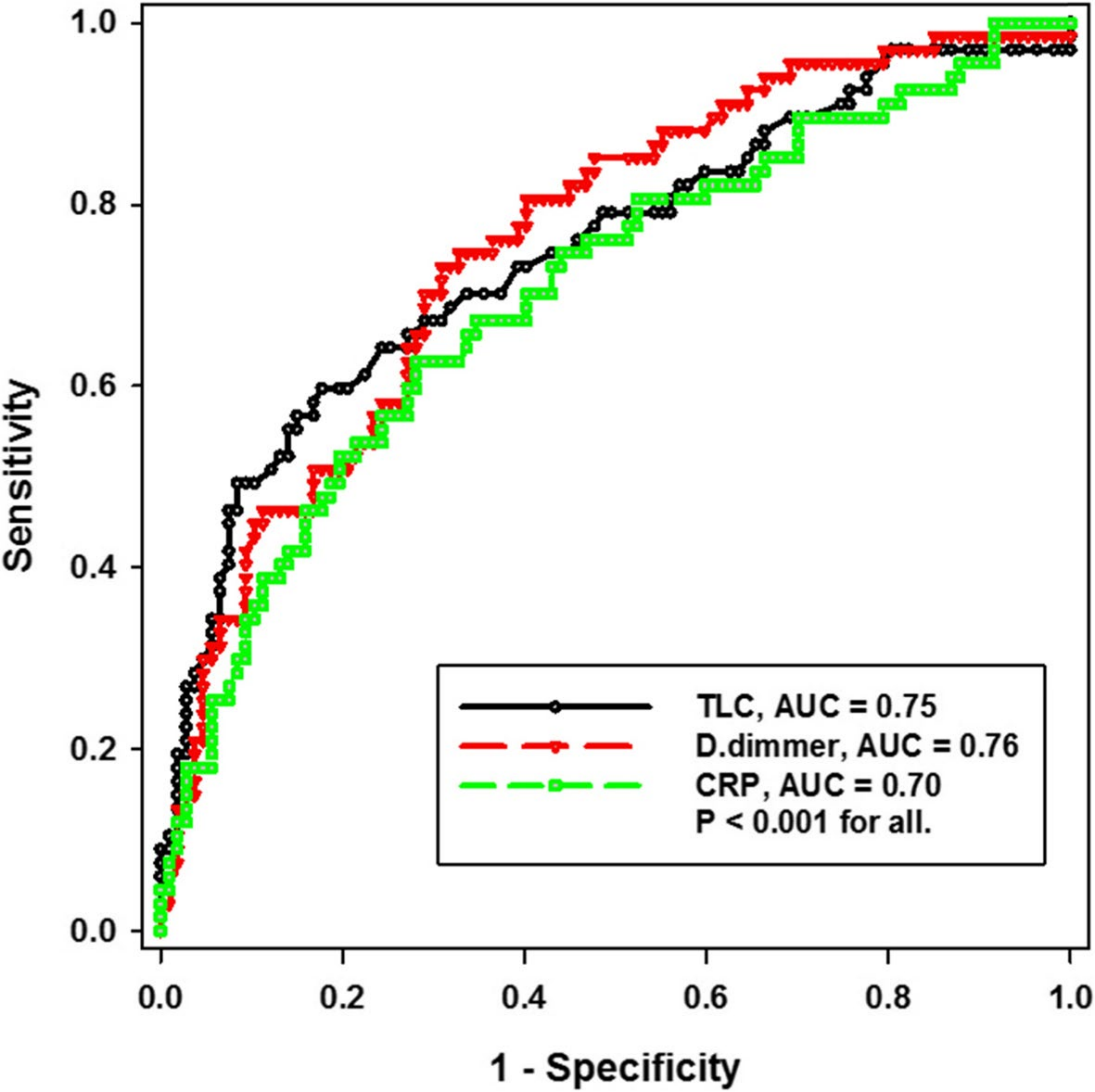
Receiver operating curve for the sensitivity and specificity of TLC, d-dimer, and CRP in predicting the severity of COVID-19 pneumonia. TLC, total leukocytic count; AUC, area under the curve; CRP, C-reactive protein

In multivariate regression, only leukocytosis (adjusted odds ratio [aOR] 1.14, 95% confidence interval [CI] 1.1, 1.2), elevated d-dimer (adjusted odds ratio [aOR] 1, 95% confidence interval [CI] 1.0001, 1.0004), and elevated CRP (adjusted odds ratio [aOR] 1.01, 95% confidence interval [CI] 1.0015, 1.0103) were found to be independent predictors of severe disease (Fig. 1; Table 3).

**Table 3:** Accuracy of elevated TLC, d-dimer, and CRP in predicting COVID-19 pneumonia severity

Predictor	Cutoff	AUC	Sensitivity (%)	Specificity (%)	PPV (%)	NPV (%)	*P* value
TLC (× 10^3^/μl)	12.9	75	61	78	37	90	< 0.001
d-dimer (μg/ml)	1141	76	70	70	34	91	< 0.001
CRP (mg/l)	97.65	70	67	65	30	90	< 0.001

*TLC* Total leukocytic count, *CRP* C-reactive protein, *PPV* Positive predictive value, *NPV* Negative predictive value, *AUC* Area under the curve

### Factors associated with in-hospital mortality

The mortality rate in our cohort was 19.4%, which was significantly associated with old age and comorbidity, especially renal disease, *P* = 0.002, 0.02, and < 0.001, respectively. Moreover, patients with severe disease and those who needed NIV and IMV for oxygen supply had a significantly higher mortality rate, *P* < 0.01 for all. Elevated levels of TLC, AST, ALT, urea, creatine, ferritin, d-dimer, and CRP, as well as lower levels of lymphocyte, platelet, and hemoglobin, were found to be significantly associated with mortality (Table 2).

**Fig. 2:**
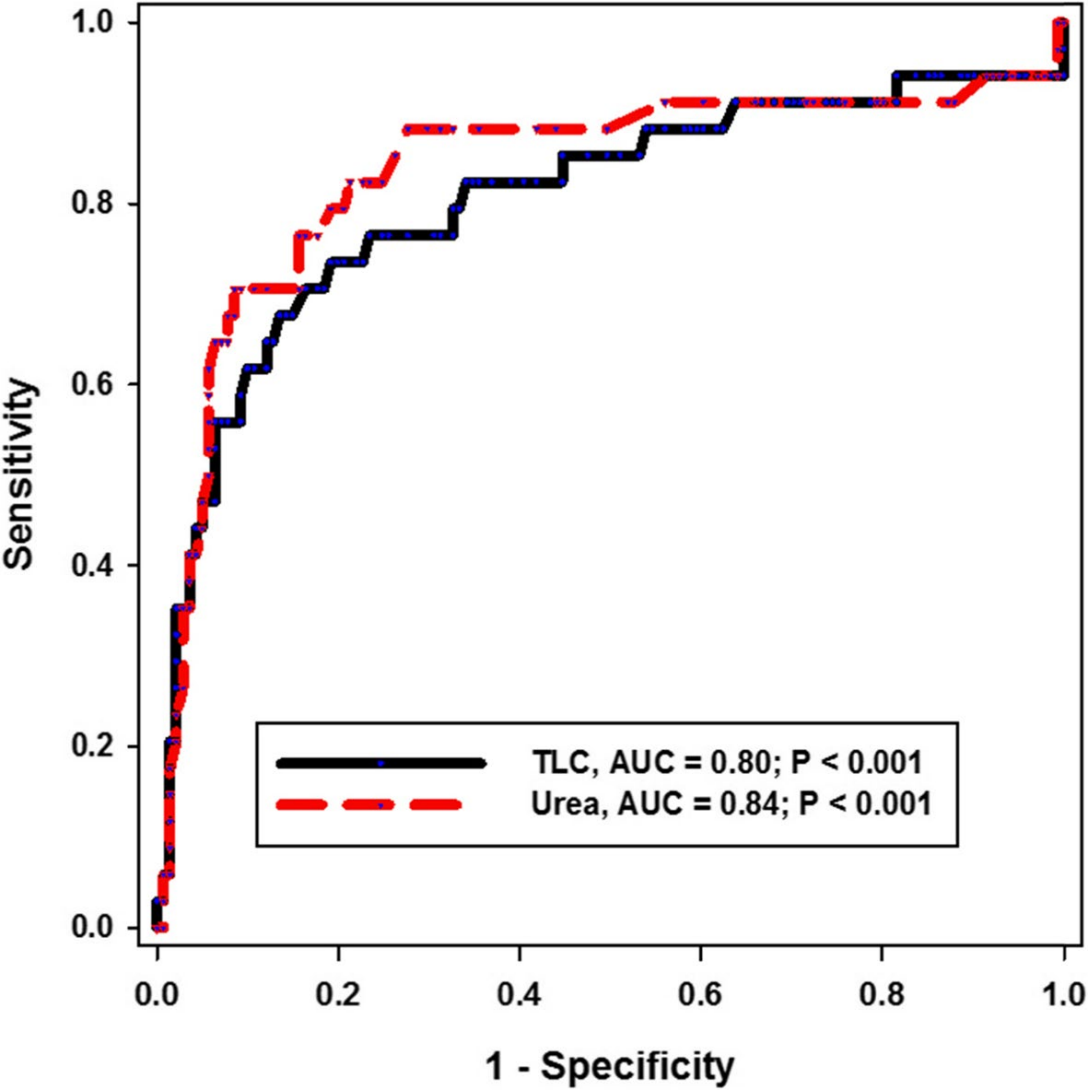
Receiver operating curve for the sensitivity and specificity of TLC and urea in predicting the in-hospital mortality of COVID-19 pneumonia. TLC, total leukocytic count; AUC, area under the curve

Multivariate analysis was performed to identify the independent predictors for mortality. Elevated TLC (adjusted odds ratio [aOR] 1.17, 95% confidence interval [CI] 1.005, 1.363), urea (adjusted odds ratio [aOR] 0.99, 95% confidence interval [CI] 0.974, 0.997), and the use of invasive mechanical ventilation (adjusted odds ratio [aOR] 1597.5, 95% confidence interval [CI] 60.112, 42,458.58), and presence of respiratory bacterial respiratory co-infection adjusted (odds ratio [aOR] 71.2, 95% confidence interval [CI] 1.5, 3381.9) were found to be independently associated with mortality (Fig. 2; Tables 4 and 5).

**Table 4:** Univariate Cox analysis of COVID-19 risk factors for mortality

Demographic character	Improved (*n* = 141)	Died (*n* = 34)	*P*
Age	57.085 ± 13.3	66.265 ± 15.18	**0.002**^**ƪ**^
Sex			
Female^a^	34 (24.1%)	5 (14.7%)	0.21^$^
Male^a^	107 (75.9%)	29 (85.3%)	
Comorbidity^a^	71 (50.35%)	25 (73.5%)	**0.02**^**$**^
DM^a^	50 (35.46%)	13 (38.2%)	0.84^$^
HTN^a^	46 (32.62%)	17 (50%)	0.05^$^
IHD^a^	5 (3.55%)	3 (8.8%)	0.18^$^
Malignancy^a^	4 (2.84%)	3 (8.8%)	0.13^$^
Renal disease^a^	5 (3.55%)	8 (23.5%)	**< 0.001**^**$**^
Respiratory co-infection	6 (4.26%)	13 (38.24%)	**< 0.001**^**$**^
Severe/critical^a^	37 (26.2%)	30 (88.2%)	**< 0.001**^**$**^
Oxygen use^a^	76 (53.9%)	34 (100%)	**< 0.001**^**$**^
Standard oxygen^a^	74 (52.48%)	17 (50%)	0.84^$^
HFNC^a^	22 (15.6%)	10 (29.4%)	0.06^$^
NIV^a^	7 (4.9%)	8 (23.5%)	**0.001**^**$**^
IMV^a^	4 (2.8%)	32 (94.12%)	**< 0.001**^**$**^
Admission labs			
TLC (× 10^3^/μl)^b^	10 (6.9–13)	17.9 (13.1–24.2)	**< 0.001**^**§**^
Lymphocyte (/μl)^b^	1650 (1218–2267)	1093 (765–1476)	**< 0.001**^**§**^
HB (g/dl)^c^	13.34 ± 1.88	12.547 ± 2.054	**0.03**^**ƪ**^
PLT (× 10^3^/μl)^b^	319 (235.5–407)	272.5 (187.5–347.5)	**0.009**^**§**^
AST (U/l)^b^	39 (28–57.5)	74.5 (49.2–133.2)	**< 0.001**^**§**^
ALT (U/l)^b^	50 (32–78.5)	70.5 (39.7–168.2)	**0.01**^**§**^
Urea (mg/dl)^b^	47 (39–67)	156.5 (93.2–212.2)	**< 0.001**^**§**^
Creatine (mg/dl)^b^	1 (0.9–1.3)	1.5 (0.8–3.4)	**0.008**^**§**^
d-dimer (μg/ml)^b^	711.21 (459.7–1396)	5907.4 (2414.7–8497.1)	**< 0.001**^**§**^
Ferritin (ng/ml)^b^	890 (457.5–1555.5)	2593.5 (1361.7–3913.7)	**< 0.001**^**§**^
CRP (mg/l)^b^	87.7 (34.2–157.9)	223.7 (84.7–290.3)	**< 0.001**^**§**^

*DM* Diabetes mellitus, *HTN* Hypertension, *IHD* Ischemic heart disease, *HFNC* High-flow nasal cannula, *NIV* Noninvasive ventilation, *IMV* Invasive mechanical ventilation, *TLC* Total leukocytic count, *Hb* Hemoglobin, *PLT* Platelets, *AST* Aspartate aminotransferase, *ALT* Alanine transaminase, *CRP* C-reactive protein

^ƪ^Independent t-test

^§^Mann-Whitney test

^$^Chi-square test, *P* < 0.05 considered significant

^a^Data are represented as number and percentage

^b^Data are represented as median and interquartile range

^c^Data are represented as mean and standard deviation

**Table 5:** The predictive value of elevated TLC and urea in COVID-19 pneumonia mortality

Predictor	Cutoff	AUC	Sensitivity (%)	Specificity (%)	PPV (%)	NPV (%)	*P* value
TLC (× 10^3^/μl)	16.25	80	62	90	58	91	< 0.001
Urea (mg/dl)	106	84	71	91	63	93	< 0.001

*TLC* Total leukocytic count, *AUC* Area under the curve, *PPV* Positive predictive value, *NPV* Negative predictive value

## Discussion

In this study, we provided a relatively comprehensive estimate for the early predicting factors affecting the COVID-19 disease state. We report on 175 patients with confirmed SARS-CoV-2 infection; 67 patients (38.29%) had severe and critical COVID-19 pneumonia, with the mortality rate in our cohort being 19.4%.

Multivariate regression analysis revealed that elevated CRP, d-dimer, and TLC were independent predictors of COVID-19 disease severity, whereas elevated TLC, urea, presence of respiratory bacterial co-infection, and the need for invasive mechanical ventilation were independent predictors of COVID-19 mortality.

CRP is a non-specific acute phase reactant induced by IL-6 in the liver. Elevated CRP levels are directly correlated with the level of inflammation and disease severity. A meta-analysis conducted by Malik et al. revealed that higher CRP levels are associated with disease severity and the formation of lung lesions in the early stages of COVID-19 [2]. Elshazli et al. found CRP to be a valid biomarker of death from COVID-19 when examining a range of hematological and immunological markers [3]. Elevated CRP may not be attributable to COVID-19 alone and may represent concomitant pathology such as secondary bacterial pneumonia [4].

Regarding the d-dimer levels, they were significantly higher in patients with severe COVID-19, whereas mortality was significantly associated with elevated d-dimer levels. Since the emergence of COVID-19, several data have reported that elevated d-dimer is more prevalent in deceased patients, and increasing odds of in-hospital death were associated with elevated d-dimer levels [5–7]. This finding is attributed to severe virus infection that developed into sepsis and induced coagulation dysfunction. Also, the increase of d-dimer may be an indirect manifestation of inflammatory reaction, as inflammatory cytokines could cause the imbalance of coagulation and fibrinolysis in the alveoli, which may activate the fibrinolysis system and then increase the level of d-dimer [8].

However, evidence regarding the causal mechanisms and whether the associations are specific effects of SARS-CoV-2 infection or are consequences of systemic inflammatory response is still lacking.

With respect to TLC, the present study revealed that higher TLC was associated with severe COVID-19 infection and higher mortality. Yuan et al. reported similar findings in severe COVID-19 cases [9]. Zhao et al. evaluated 52 COVID-19 patients with increased leukocyte at admission and compared them with COVID-19 patients with non-increased leukocyte count, and it was found that the patients with increased leukocyte count were more likely to develop critical illness (*P* < 0.01) and had a higher rate of death (*P* < 0.01) [10].

This finding could be explained due to high levels of serum IL-6, which induce an inflammatory response leading to neutrophil migration, recruitment, and activation. Phagocytosis, the release of granular contents, and the production of cytokines are significant functions of activated neutrophils, suggesting a protective immune response against the virus. However, excessive neutrophils can cause cytokine storm and tissue damage, leading to severe COVID-19 pneumonia and death [10].

Overall, data obtained from the multivariate analysis revealed that a higher mortality rate is expected in patients suffering from renal impairment, which could be attributed to lowered immunity and the underlying immune responses in patients with comorbid conditions [11, 12]. As evidenced by the reduction in nitric oxide in diseases such as hypertension, diabetes, and kidney dysfunction, endothelial dysfunction is also thought to be a key factor [13–15].

Henry and Lippi found over 3-fold higher risk of developing severe COVID-19 in patients suffering from chronic renal disease [16].

Tian et al. and Cheng et al. reported that the presence of chronic renal disease is a significant predictor of mortality in COVID-19 patients [6, 17]. Therefore, paying more attention to the presence of renal impairment at the time of admission and implementing an effective intervening strategy handling it as early as possible might help reduce mortality in COVID-19 patients suffering from underlying kidney disease.

It is well known that viral respiratory infections predispose patients to bacterial infections and that co-infections have a worse outcome than that of either infection on its own [18]. Different studies have addressed the presence of documented respiratory co-infection in COVID-19 pneumonia patients with varying degrees of prevalence, which may be due to different diagnostic methods applied to diagnose respiratory co-infections [19–21].

In the current study, 10.9% of our patients have documented respiratory co-infection in whom mortality was significantly increased, which is consistent with a recent meta-analysis in which investigators have shown a positive association between co-infection and increased risk of death among patients with the SARS-CoV-2 infection [22].

The use of invasive mechanical ventilation was also found to be an independent predictor of mortality in our study, which is compatible with Manal et al., who found that the need to ventilate a COVID-19 patient mechanically is likely associated with higher mortality [23].

There was a significant range of mortality rates reported for COVID-19 patients receiving mechanical ventilation ranging from 9.4 to 97% [24]. Studies from China reported mortality in COVID-19 patients receiving IMV reaching as high as 97% [7].

The wide variation in mortality rates among patients receiving invasive mechanical ventilation (IMV) could be multifactorial in different countries. Studies from different areas with varying degrees of expertise and hospital resources may explain the variable reported outcome. In addition, during the surge of the pandemic, different institutional protocols were applied according to the available resources, like in some Italian regions, giving priority to patients who are more likely to survive, as was recommended by the Italian Society of Anesthesia, Analgesia, Resuscitation and Intensive Care [25, 26].

Studies from China early in the pandemic had reported higher mortality rates than US, UK, and Spanish studies that recruited patients later in the pandemic [27–31].

In other words, decreasing mortality rates as the pandemic progresses have been observed in studies including ICU COVID-19 patients and in which the proportion of patients receiving IMV ranged from 0 to 100%. This finding could explain why clinicians gained more knowledge and expertise as time passed, and medical treatments became more available compared to early in the pandemic [24].

Our study has some limitations. First, due to the retrospective study design, not all laboratory tests were done in all patients, including lactate dehydrogenase and IL-6. Therefore, we could not investigate their role in predicting the outcome in COVID-19 patients. Second, the study was performed in a limited hospital setting and included a relatively small sample size with disproportion in the different study groups. In order to validate our findings, a large multicenter prospective observational study would be preferable.

In conclusion, according to this study, COVID-19 infection was more aggressive in patients presenting with elevated TLC, d-dimer, and CRP levels. Mortality was found to be higher in patients with renal impairment and documented respiratory co-infection as well as in patients with elevated TLC and mechanically ventilated patients.

Hence, pretreatment clinical and laboratory data from COVID-19 patients at hospital admission can assist clinicians in identifying high-risk patients early and providing special and prompt care for those in need.

## Data Availability

Data are available.

## Abbreviations

COVID-19: Coronavirus disease of 2019
SARS-CoV-2: Severe acute respiratory syndrome coronavirus 2
CRP: C-reactive protein
TLC: Total leukocytic count
WHO: World Health Organization
RT-PCR: Reverse transcription polymerase chain reaction
ROC: Receiver operating characteristic curve
AUC: Area under the curve
DM: Diabetes mellitus
HTN: Hypertension
HFNC: High-flow nasal cannula
NIV: Noninvasive ventilation
AST: Aspartate aminotransferase
ALT: Alanine transaminase
IL-6: Interleukin-6
IMV: Invasive mechanical ventilation
ICU: Intensive care unit
